# Exploring socioeconomic inequities in access to palliative and end-of-life care in the UK: a narrative synthesis

**DOI:** 10.1186/s12904-021-00878-0

**Published:** 2021-11-21

**Authors:** Maddy French, Thomas Keegan, Eleftherios Anestis, Nancy Preston

**Affiliations:** 1 Division of Health Research, Lancaster University, Lancaster, UK; 2 Lancaster Medical School, Lancaster University, Lancaster, UK

**Keywords:** Socioeconomic position, Healthcare utilisation, Access to healthcare, Palliative care, End-of-life care

## Abstract

**Background:**

Efforts inequities in access to palliative and end-of-life care require comprehensive understanding about the extent of and reasons for inequities. Most research on this topic examines differences in receipt of care. There is a need, particularly in the UK, for theoretically driven research that considers both receipt of care and the wider factors influencing the relationship between socioeconomic position and access to palliative and end-of-life care.

**Methods:**

This is a mixed studies narrative synthesis on socioeconomic position and access to palliative and end-of-life care in the UK. Study searches were conducted in databases AMED, Medline, Embase, CINAHL, SocIndex, and Academic Literature Search, as well as grey literature sources, in July 2020. The candidacy model of access, which describes access as a seven-stage negotiation between patients and providers, guided study searches and provided a theoretical lens through which data were synthesised.

**Results:**

Searches retrieved 5303 studies (after de-duplication), 29 of which were included. The synthesis generated four overarching themes, within which concepts of candidacy were evident: identifying needs; taking action; local conditions; and receiving care.

**Conclusion:**

There is not a consistent or clear narrative regarding the relationship between socioeconomic position and receipt of palliative and end-of-life care in the UK. Attempts to address any inequities in access will require knowledge and action across many different areas. Key evidence gaps in the UK literature concern the relationship between socioeconomic position, organisational context, and assessing need for care.

**Supplementary Information:**

The online version contains supplementary material available at 10.1186/s12904-021-00878-0.

## Background

There have long been concerns that access to healthcare is influenced not only by an individual’s need for care but also by their position in society relative to others [1, 2]. One indicator of relative position is a person’s socioeconomic position, typically derived from level of income, education, or employment, through which individuals obtain skills, knowledge, and assets that allow them to benefit from society [3]. These individual-level indicators derive from social structures and as such, socioeconomic position cannot be divorced from the wider society in which people live. It is both the product of how society is structured and how individuals act within the constraints of society [4].

Socioeconomic position has long been associated with health outcomes, with people in a more disadvantaged socioeconomic position nearly always experiencing poorer health [3, 5]. While improving access to healthcare is not sufficient on its own to overcome these differences, fair access across socioeconomic groups is a vital component of an equitable healthcare system, facilitating opportunities to improve health outcomes [6]. There is a drive within palliative and end-of-life care to understand and improve inequities in access, including between socioeconomic groups [7–9]. Comprehensive understanding about the extent and nature of socioeconomic inequities in accessing this care is critical to these efforts.

Most research on socioeconomic inequities in accessing palliative and end-of-life care examines differences in receipt of care, often indicating an association between socioeconomic disadvantage and a lower likelihood of receiving specialist palliative care [10–13]. Conversely, use of hospital-based care in the last year of life tends to be higher for those in a more disadvantaged socioeconomic position, with poorer health likely accounting for some of this use [8, 14, 15]. However, receipt of care is only one component to accessing care. Access also refers to how people and healthcare professionals identify needs, navigate services, and express preferences, all of which takes place in the context of local service availability, ultimately leading to offers of care being made and potentially rejected [16]. Despite being critical to understanding whether receipt of care is inequitable, fewer studies have explored the relationship between socioeconomic position and these wider components of access.

One review, now 10 years old, suggested that inequitable use of palliative care in high income countries may partly relate to the geographic inaccessibility of services in disadvantaged areas [11]. The review also highlighted issues around mistrust, lower levels of health literacy and communication difficulties between healthcare providers and those experiencing socioeconomic disadvantage, drawing largely on evidence from the United States, and to a lesser extent the UK [11]. Services, and the people providing them, may also stigmatise some patients experiencing social disadvantage, or not facilitate choices that are desirable or realistic given the circumstances of people’s lives [17, 18].

There is justification for examining socioeconomic position and access to palliative and end-of-life care in the UK context. Most evidence relating to socioeconomic position and access to palliative and end-of-life care comes from the United States. A meta-analysis of studies examining the association between area deprivation and use of specialist palliative care, for example, pooled results from 24 studies from three countries, with 14 studies from the United States, six from Canada, and none from Europe [10]. While some findings are likely to be transferable between countries, evidence from countries with insurance-based healthcare systems is not easily applied to those providing universal, or close to universal, healthcare. The regulations and financial reimbursement related to hospice and specialist paliative care referrals in some countries may create barriers to access not found in the UK, for example [19].

Interventions to improve outcomes in palliative care, and which aim to support people in socioeconomically disadvantaged circumstances, are likely to be more effective when the context in which they are delivered is considered [20, 21]. In order to address any socioeconomic inequities in access to palliative care, policy makers and practitioners must understand the barriers to access related to the organisational and socioeconomic context specific to that setting. However, there is a paucity of theory-driven research in this area, which could help identify which aspects of a healthcare system or country setting hinder or facilitate access for those experiencing socioeconomic disadvantage.

There is a need for theoretically driven research that considers the importance of local context for understanding the relationship between use of palliative care and socioeconomic position. In this review, evidence relating to socioeconomic position and access to palliative and end-of-life care is closely examined in a single country (the United Kingdom), looking both at receipt of care and the factors influencing it. Focusing on a single country allows evidence to be examined without having to account for between country differences, and will help identify access-influencing factors for which there is good or poor evidence in the UK.

### Palliative and end-of-life care in the UK

Palliative and end-of-life care in the UK is provided by a mixture of state-funded NHS services and predominantly voluntary-funded hospice organisations. The formal provision of palliative care by healthcare services has grown substantially in the country since the 1960s, when it was developed around the care of people with cancer as part of the modern hospice movement [22]. Today, the UK has one of the most well-developed and high quality palliative care sectors in the world [23]. Recent developments include a push towards generalist palliative care, encouraging a view of palliative and end-of-life care as something that should be widely available across the healthcare system, and not just in a small number of specialist inpatient units predominantly focused on patients with cancer [24]. However, estimates suggest that around half a million people could need palliative care in the UK by 2040, likely necessitating changes to palliative care models to accommodate this growing need [25].

### The candidacy model of access

In this review, the suitability of an existing theoretical model to understand issues of access to palliative and end-of-life care is explored, with the potential for this model to be applied to other countries and settings. A model of healthcare access arguably relevant across settings and countries is the candidacy model of access [16]. Developed from an analysis of healthcare access for people experiencing socioeconomic disadvantage, the candidacy model of access proposes seven stages of negotiation between patients and providers that are potentially influenced by socioeconomic position: identification of candidacy; navigation of services; permeability of services; appearances; adjudications; offers or resistance; and operating or local conditions (Table 1). The model has been applied to diverse care settings, including public services [26], maternity services in resource-poor settings [27], and mental health care [28], but to our knowledge has not been applied to palliative and end-of-life care. It is used in this review to guide the study searches and provide a theoretical analytical framework.

**Table 1:** The stages of candidacy

Stages of candidacy	Description
Identification of candidacy	The process by which people recognise their symptoms need medical attention or intervention.
Navigation	The work people have to do in order to use services.
Permeability of services	Describes how permeable a service is. A permeable service is one that is easy to use and does not involve gatekeeping, for example through referral procedures. Also requires cultural alignment between users and services.
Appearances at health services	The way in which people appear to service providers and how they assert a claim to candidacy for medical attention.
Adjudication	The professional judgements made about candidacy and the influence these have on the ongoing care of patients.
Offers and resistance	The pattern to which offers are made by professionals and resisted by patients.
Operating conditions and the local production of candidacy	The locally specific influences on interactions between professionals and patients.

### Review aims

Two key questions are explored in this review: (1) to what extent is socioeconomic position associated with access to palliative and end-of-life care in the UK and (2) how do factors relating to socioeconomic position influence access to this care. A further aim is to explore the usefulness of the candidacy model for understanding socioeconomic inequities in access to palliative and end-of-life care. Consequently, the review includes evidence on both receipt of care and the wider factors influencing access.

## Methods

This review took the form of a narrative synthesis, using text rather than statistics to convey the meaning of the data from primary studies [29]. The review process was guided by the four stages of a narrative synthesis (Table 2).

**Table 2:** Stages of a narrative synthesis [29]

Stages of narrative synthesis	This synthesis
Stage 1: Developing a theory of how the intervention works, why and for whom.	The candidacy theory of accessing healthcare [16] provided a theoretical model for understanding access to palliative and end-of-life care for people experiencing socioeconomic disadvantage. Prior to conducting the synthesis, the model was adapted to incorporate additional factors related to palliative and end-of-life care (Supplementary Material 1).
Stage 2: Developing a preliminary synthesis of findings of included studies.	Initial coding was carried out using pre-defined and open coding. Some studies were grouped by characteristics to try to identify patterns in the data.
Stage 3: Exploring relationships in the data	Text summaries and concept mapping techniques were used to link findings and find reoccurring themes. Data were explored under the seven stages of candidacy to examine how they fitted to the model.
Stage 4: Assessing the robustness of the synthesis.	Hawker et al.’s [30] critical appraisal tool was used to assess the quality of the primary studies.

### Study searches

A comprehensive search of the literature was undertaken in July 2020 to find relevant English language, peer-reviewed articles and grey literature reports. Search terms and subject headings relating to palliative care, access, and socioeconomic position were combined with AND in searches on journal databases AMED, Medline, Embase, CINAHL, SocIndex, and Academic Literature Search. Searches were conducted in these databases on 6th July 2020. Search terms were developed with reference to Cochrane guidance on finding palliative care literature [31] and the original literature synthesis that generated the candidacy model of access [16]. An example search strategy (Medline) is included in Supplementary Material 2. Reference lists and citations of systematic reviews and included studies were also searched. The websites of the relevant governmental health departments in the UK, and of charities Hospice UK and Marie Curie, were searched for grey literature.

### Study screening

The study population included patient population groups with any advanced progressive illness, their families, healthcare professionals, or organisations providing their care in the UK. Studies using any methods to research socioeconomic position and access to palliative or end-of-life care for the study population specified above were eligible. Studies that looked at palliative care, end-of-life care, people in the last year of life, or hospice care in any setting were eligible for inclusion. This means studies of care in both generalist (e.g. hospitals, primary care) and specialist (e.g. hospices) palliative care settings were included. Studies were included if they referenced in the title or abstract the indicator of socioeconomic position used. Studies that considered social characteristics (e.g. gender, age, ethnicity) were only included when social characteristics were linked to economic characteristics (e.g. income, deprivation, occupational status).

Studies published prior to 1990 were excluded, as prior to this time many palliative care providers in the UK were in the early stages of development and had only just become established [32]. Additionally, studies that only considered place of death were excluded. While other studies have included place of death as an indicator of access [12], and there is some evidence to suggest use of specialist palliative care may help mitigate the effect of socioeconomic position on place of death [33], place of death is a potentially misleading indicator of access. This is because of the diversity in individual patients’ preferences for dying at home [34], challenging the assumption that all deaths in hospital indicate poor access to care. More recent evidence suggests that increasing use of hospital services at the end of life by people in a more disadvantaged socioeconomic position is partly explained by poor health [14], suggesting reasons beyond access to care may influence the likelihood of dying in hospital.

A full list of inclusion and exclusion criteria is provided in Supplementary Material 3. Titles and abstracts of all retrieved studies were screened against the eligibility criteria (MF); 10% of these studies were screened by a second reviewer (EA) and disagreements were resolved in further discussions about the inclusion criteria.

**Fig. 1:**
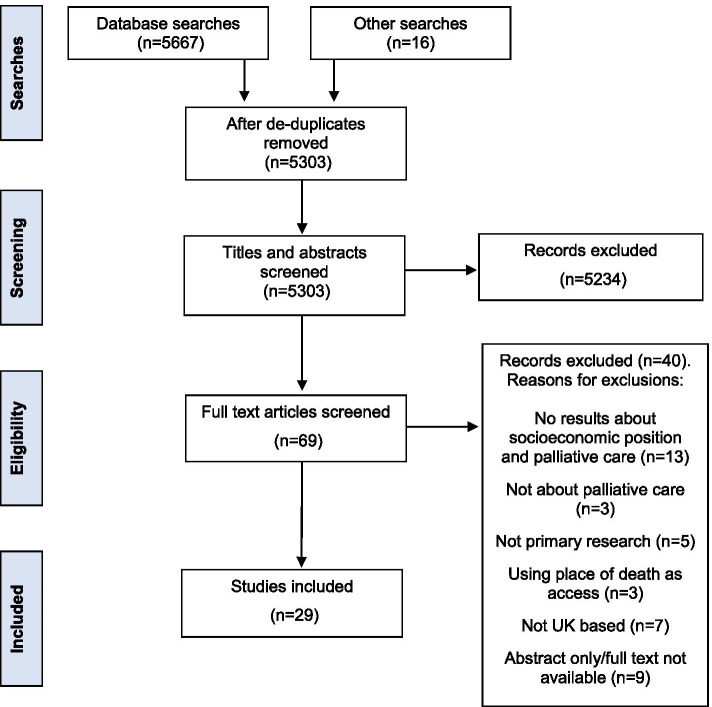
PRISMA flow diagram of study screening

MF retrieved and screened the full texts of 69 studies and a further 40 were excluded (Fig. 1) resulting in 29 included studies. The characteristics of included studies were input into a spreadsheet using a data extraction form (Supplementary Material 4); quality appraisal was carried out at the same time using Hawker et al.’s [30] critical appraisal tool (Supplementary Material 5), chosen for this review because of its appropriateness for both qualitative and quantitative studies. Included studies were scored (1 – very poor, 2 – poor, 3 – fair, 4 – good) for each of the nine quality domains in the tool, aggregated into an overall study score on the conduct and reporting of the study. All studies were appraised for quality by MF and 10% were appraised by EA. Domain scores for compared studies were similar from each reviewer, with no more than a one point difference in each domain, resulting in no more than a four-point difference in the overall study scores. There was no quality score threshold for exclusion, although where studies reported contradictory findings, findings from the higher quality study were emphasised in the reporting of the synthesis results.

### Synthesising data

The analysis followed the stages of a narrative synthesis, drawing on both inductive and deductive approaches (Table 2). MF developed a preliminary synthesis (stage 2) by coding the result sections of quantitative studies looking at *receipt of care* in Nvivo. Codes were then grouped by study characteristics to try to identify trends in findings. Subsequently, the results and discussion sections of quantitative and qualitative studies that examined *other access issues* were coded by MF using open coding and pre-defined codes from the candidacy framework [16].

The relationships between findings were explored (stage 3) using concept maps, whereby codes and themes from qualitative and quantitative evidence are diagrammatically displayed to help establish links between them [29]. Findings relating to the candidacy concepts were summarised by text before returning to the primary sources to identify any further data. These text summaries were rewritten, incorporating further findings and synthesising ideas into the final themes.

### Assessing the robustness of the synthesis

Efforts were made to increase the robustness of the synthesis by using two reviewers to select and screen studies, helping to clarify study eligibility and ensure rigour in the assessment of study quality. Viewing findings through the lens of an existing theory of access also helped to incorporate concepts relevant to accessing palliative care not previously considered by the authors.

## Results

Searches retrieved 5303 studies (after de-duplication), 29 of which have been included in this review. Tables 3 and 4 describe the characteristics of studies with findings relating to receipt of palliative and end-of-life care (Table 3) and findings related to other access issues (Table 4). Some studies contributed multiple findings and are included in both tables. The studies ranged widely in purpose and varied in quality, with scores ranging from 17 to 35; most studies scored between 25 and 30.

**Table 3:** Findings relating to receipt of specialist and generalist palliative care

Author(s)	Care setting	Aims	Method	Socioeconomic measure	Population	Key findings relating to review questions
*Specialist palliative care*						
Addington-Hall et al., 2000 [35] Quality score 33	Community services (specialist palliative care)	Understand how cancer patients who received community specialist palliative care differ from those who did not.	Survey	Social class (I-V) No reference or details provided.	Patients (proxy)	Social class was not statistically significantly associated with receipt of community specialist palliative care.
Addington-Hall and Altmann, 1998 [36] Quality score 30	Inpatient hospice	Understand how cancer patients who received hospice inpatient care differed from those who do not.	Survey	Social class (I-V). Coded from occupations on death certificates.	Patients (proxy)	Social class was not statistically significantly associated with receipt of hospice inpatient care.
Allsop et al., 2018 [37] Quality score 34	Inpatient and community hospice care	Understand how patient and organisational factors influence the duration of hospice-based palliative care prior to death.	Routine data	Geographic regions (North, South, Midlands)	Patients/Hospices	Looked at timing of referral. On average, hospices in the North of England had a shorter median number of days between referral and death than those in the Midlands, London and South of England.
Buck et al., 2018 [38] Quality score 28	Hospice at home	Describe the care provided by a hospice at home service.	Routine data	Area deprivation (Index of Multiple Deprivation)	Patients	Smaller proportion of referrals came from most deprived area. Deprivation scores of those who received care were significantly lower (less deprived) than those of general population in all but one area.
Burt et al., 2010 [39] Quality score 35	Outpatient and community specialist palliative care	Understand the effect of age on use of services after accounting for need, including area deprivation.	Routine data	Area deprivation (Index of Multiple Deprivation)	Patients	No statistically significant association between receipt of specialist palliative care and area deprivation.
Campbell et al., 2010 Quality score 32 [40]	Hospice at home	Explore how socioeconomic position influences access to hospice at home.	Routine data	Area measures: Deprivation (Index of Multiple Deprivation) Educational attainment Approximated Social Grade Economic activity Household tenure	Patients	Suggests that socioeconomic characteristics not service provision or cancer mortality predicts ward-level referral rate, including area measures of deprivation, social grade, and economic activity.
Cartwright, 1992 [41] Quality score 22	Inpatient hospice (specialist palliative care)	(i) Understand the impact of social differences in mortality on life before death; and (ii) examine the extent to which experiences differ between social groups in this time.	Survey	Social class (I-V) Definitions from 1980 Classification of Occupations.	Patients (proxy)	More middle class patients admitted to private hospital or hospice than working class.
Dixon, et al., 2015 [7] Quality score 31	Community services (specialist palliative care)	Identify and explore systematic differences in access or outcomes, between geographical areas, settings or different groups of service-users.	Survey	Area deprivation (Index of Multiple Deprivation)	Patients (proxy)	No evidence of a difference in receipt of care from Marie Curie Nurses or hospice at home services between areas of deprivation
Gray and Forster, 1997 [42] Quality score 29	Any specialist palliative care	(i) Provide district with information about their current service provision; and (ii) inform national debate about use of specialist palliative care services.	Routine data	Social class (I-V) based on Office of Population Censuses and Surveys (OPCS) occupational classifications	Patients	The majority of cases in both groups (received/did not receive care) were in the lower social classes (Mm, IV and V). There were no significant differences regarding receipt of care.
Johnson et al., 2018 [43] Quality score 32	Any specialist palliative care	Investigate whether access to specialist palliative care services ameliorates the effects of respondents’ socioeconomic position on decedents place of death. Study reports differences in access by respondent income.	Survey	Household income Qualifications	Family caregiver Patients (proxy)	**Income:** No evidence of association between income of respondents and access to care. Income was missing for 20% of respondents. **Qualifications:** When respondents had a degree, decedents were statistically significantly more likely to access care. Nearly 100% provided qualification status.
London Cancer Alliance, PallE8 and Marie Curie (London Cancer Alliance) 2015 [44] Quality score 26	Inpatient and community hospice care	Understand more about the provision of specialist palliative care in London	Routine data	Area deprivation scoring taken from 2011 London Cancer Alliance’s audit exercise	Clinical Care Commissioning Groups (CCGs)	**Community:** Some CCGs in North East London had high relative deprivation scores and comparatively lower proportion of people with cancer who accessed community services. **Inpatient:** An association with deprivation was less apparent with inpatient units. Statistical significance not reported.
Marie Curie Cancer Care and the Bevan Foundation (Marie Curie) 2014 [8] Quality score 25	All specialist palliative care	Explore whether access to palliative care services may be shaped by people’s socio-economic status, exacerbating existing inequalities in the incidence of diseases, as well as by factors such as age and diagnosis.	Routine data	Area deprivation (no details on index used)	Patients	Nearly the same proportion in most and least deprived areas received care. For deaths from cancer, the proportion of people receiving specialist palliative care is slightly higher in most deprived quintile. Statistical significance not reported.
*Generalist palliative care*						
Cartwright, 1992 [41] Quality score 22	GP and home nursing (generalist palliative care)	(i) Understand the impact of social differences in mortality on life before death; and (ii) examine the extent to which experiences differ between social groups in this time.	Survey	Social class (I-V) Definitions from 1980 Classification of Occupations.	Patients (proxy)	No class difference in home visits from GP (adjusted for age) or receipt of home nursing help.
Dixon, et al., 2015 [7] Quality score 31	Community services (generalist palliative care)	Identify and explore systematic differences in access or outcomes, between geographical areas, settings or different groups of service-users.	Survey	Area deprivation (Index of Multiple Deprivation)	Patients (proxy)	No evidence of a difference in receipt of care from rapid response teams or ‘other’ nurses between areas of deprivation.
Fisher et al., 2016 Quality score 26 [45]	Out of hours (generalist palliative care)	Describe patterns of usage of patients presenting to the out-of-hours service and labelled by the service as ‘palliative’.	Routine data	Area deprivation (Index of Multiple Deprivation)	Patients	Patients contacting the service with palliative needs lived in relatively less deprived area than contacts for all other causes.
Grande et al., 2002 [46] Quality score 32	Hospital at home (last 2 weeks of life)	Understand differences between patients receiving hospital at home and not, in terms of their overall healthcare use.	Routine data	Area deprivation (Jarman underprivileged area; Townsend Index) Social class derived from Standard Occupational Class (SOC).	Patients	**Area deprivation:** Patients referred to hospital at home came, on average, from less deprived areas than those who were not referred to hospital at home. **Social class:** No statistically significant difference in referrals by social class.
Hanratty et al., 2008 [15] Quality score 23	Hospital (end-of-life care)	Explore the value of linked mortality and hospital activity data in palliative care research (by exploring the relationship between deprivation and hospital stays at end of life).	Routine data	Area deprivation (Carstairs index)	Patients	Use of hospital services in the last year of life varied by area deprivation for patients with cancer and heart failure. Residents of the most deprived areas with heart failure were more likely than patients from other areas to spend more days in hospital. Patients with cancer from the most deprived areas were more likely to be admitted frequently but less likely to be amongst the longest staying patients.
Hanratty, Jacoby, and Whitehead, 2008 [47] Quality score 32	GP services	(i) Analyse use of and payment for health and welfare services in the year before death for decedents in different financial circumstances; and (ii) determine their receipt of relevant illness related state benefits.	Survey	Perception of financial circumstances. Annual household income.	Patients	People who reported financial difficulties had more than an 80% increase in the likelihood of being a frequent attender of GP services and were less likely to pay for services. Paying for care was also associated with high use of GP services.

**Table 4:** Findings relating to other access issues

Author(s)	Care setting	Aims	Method	Socioeconomic measure	Population	Key findings relating to review questions
Barclay et al., 2003 [48] Quality score 32	GP services	Compare palliative care training of GPs in deprived south Wales valleys with rest of Wales.	Survey	Geographic regions	HCP	**Access issues:** How HCP assessed patients. **Key findings:** There was no evidence of a difference between GPs in terms of palliative care training in areas of high and low social deprivation at any of the four career stages. GPs in the more deprived region were older, longer qualified and more likely to be non-UK graduates.
Cartwright, 1992 [41] Quality score 22	Inpatient hospice services (specialist palliative care) GP and home nursing (generalist palliative care)	(i) Understand the impact of social differences in mortality on life before death; and (ii) examine the extent to which experiences differ between social groups in this time.	Survey	Social class (I-V) Definitions from 1980 Classification of Occupations.	Patients (proxy)	**Access issues:** Symptom burden, patient resources, patient awareness, patient need. **Key findings:** More middle class had good quality of life in last year. More working class reported problems with costs of keeping home warm, adapting house to needs. More working class had financial problems. No class difference in symptoms apart from more dry mouth reported by working class; no difference in awareness of dying or being able to find all information wanted.
Clark, 1997 [49] Quality score 17	Hospice at home	Describe the use of a hospice at home service.	Routine data	Area deprivation (Jarman index)	Patients	**Access issues**: Service capacity **Key findings:** Patients living in the more deprived areas received twice as many visits at home as those in the less deprived areas. Statistical significance not reported.
Dixon, et al., 2015 [7] Quality score 31	Community services (specialist and generalist palliative care)	Identify and explore systematic differences in access or outcomes, between geographical areas, settings or different groups of service-users.	Survey	Area deprivation (Index of Multiple Deprivation)	Patients (proxy)	**Access issues:** Satisfaction / unmet need for care **Key findings:** Families of deceased who lived in more deprived areas were statistically significantly less likely to say they received sufficient help and support, and to have received spiritual or emotional support.
Fergus et al., 2010 Quality score 29 [50]	Out of hours (generalist palliative care)	Identify key issues relating to out of hours care for palliative care patients, carers and professionals.	Qualitative interviews	Area measures of: Income Unemploy-ment Social grade Household type Car ownership	Patients Carers HCP	**Access issues:** patient understanding, communication, relationship with healthcare providers, resistance to care, service organisation, gatekeeping. **Key findings**: The rigmarole of access made patients reluctant to access services. Some patients misunderstood the service, assuming transfer was automatic. Bad (stressful) experiences led to decision not to contact the service again and district nurses felt it hindered contact with GPs. There was a need for better communication and information sharing to improve decisions during out-of-hours care.
Gatrell and Wood, 2012 [51] Quality score 34	Inpatient hospice (specialist palliative care)	Visualise and understand geographic patterns of both the demand for, as well as the supply of, specialist inpatient hospices.	Spatial analysis	Area deprivation (Index of Multiple Deprivation)	Hospices	**Access issues:** Geographic accessibility **Key findings:** There are 5.35 million adults living in areas of England and Wales that have higher than average deprivation and demand (cancer deaths) but below average access to inpatient hospice.
Hanratty et al., 2012 [52] Quality score 30	End-of-life care (any)	Explore people’s experiences of transitions between healthcare settings at the end of life.	Qualitative interviews	Occupational class Disadvantaged areas (Spearmen areas)	Patients	**Access issues:** Communication, relationship with healthcare providers, patient attitudes, service organisation. **Key findings:** Most participants were from disadvantaged areas and the findings may reflect issues around socioeconomic experiences. Patients reported positive experiences with individuals but challenges negotiating transitions, particularly when system priorities were not aligned with patient priorities, in securing support across settings, and communication between HCP and patients. Authors noted that findings showed little or no variation with socioeconomic status. However, socioeconomic factors were not the focus of the study.
Kessler et al., 2005 [53] Quality score 26	Hospice GP services	(i) Clarify the relationship between social class and place of death; and (ii) explore carer anxiety and barriers to control for people of a lower socioeconomic position receiving palliative care.	Qualitative interviews	Social class (I-V). Taken from Standard Occupational Classification.	Patients Carers	**Access issues:** Patient attitudes and awareness, relationship with healthcare providers; patient resources, information seeking **Key findings:** Disadvantaged social class associated with having relatives close by and more available, expressing less desire for information, and passively receiving information. Families often relied on their most forceful members, particularly children of higher social class, to help negotiate barriers to accessing care. No evidence of class differences in anxiety or attitudes towards hospice or awareness of death.
Koffman et al., 2007 [54] Quality score 31	Any palliative care Macmillan cancer (specialist palliative care)	(i) explore the awareness of palliative care and related services among UK cancer patients; and (ii) analyse the relationship between demographic factors and patients’ knowledge-base	Survey	Area deprivation (Index of Multiple Deprivation)	Patients	**Access issues:** Patient awareness and understanding. **Key findings:** Patients in the least deprived areas were 8.4 times as likely to recognise the term palliative care and 7 times as likely to correctly understand the role of Macmillan nurses, than those in the most deprived areas.
Rees-Roberts, M. et al. 2019 [55] Quality score 31	Specialist palliative care (community services)	To describe and compare the features of hospice at home services in England and understand key enablers to service provision	Survey	Area deprivation (details not provided)	Hospices	**Access issues:** Geographic accessibility **Key findings:** 7.1% of hospice at home services are provided in predominantly deprived areas, 15.7% in predominantly affluent areas, and 77.1% in mixed deprivation areas.
Seale, et al., 1997 [56] Quality score 28	NA (death awareness)	Report the prevalence of different awareness contexts and explore the causes of differences.	Survey	Social class (I -V). No reference or details given.	Patients (proxy)	**Access issues:** Patient awareness. **Key findings**: Being in a higher (I and II) social class increased the odds of someone dying in full open awareness by 2.66 times, compared to being in classes IV and V. This remained statistically significant for just cancer decedents but not non-cancer decedents. Those who died in an open awareness context were more likely to have died in a hospice.
Spruyt, 1999 [57] Quality score 22	Community-based care (all palliative care)	Increase understanding of the Bangladeshi community’s experiences of palliative care in East London.	Qualitative interviews Routine data	Not formally measured but local area described as deprived and disadvantaged.	Carers	**Access issues:** Communication, healthcare costs, healthcare quality **Key findings:** Issues with finances and re-housing influenced carers’ experiences of supporting patients and their impression of the quality of formal healthcare services.
Walsh and Laudicella, 2017 [58] Quality score 30	End-of-life care (hospital)	(i) examine whether there is a socioeconomic gradient in end-of-life healthcare costs; and (ii) whether any observed disparities are underpinned by greater use of emergency admission amongst patients in a more disadvantaged socioeconomic position.	Economic analysis	Area income deprivation (Indices of Deprivation: Income Deprivation Domain)	Patients	**Access issues:** Healthcare resources and costs **Key findings:** End-of-life healthcare costs in England are highest amongst cancer patients who live in more income deprived areas, largely due to the higher use of emergency service by these patients. The most deprived groups have longer stays in hospital after an emergency admission.
Wilson, 2009 [59] Quality score 26	Nurses (specialist palliative care) District nurses (generalist palliative care)	(i) explore whether the lifestyle factors of a patient influences nurses’ pain management decisions; and (ii) explore if post basic education and experience of pain and pain management in the clinical setting influences nurses’ attitudes in relation to pain.	Survey	Occupation	HCP	**Access issues:** How HCP assessed patients, stigmatising attitudes **Key findings:** Generalist nurses were significantly less likely to recognise the pain described by businessman than a construction worker with a history of drink driving.
Wood et al., 2004 Quality score 28 [60]	Inpatient hospice (specialist palliative care)	Assess the extent to which those living in particular wards in North West England have equity of access to adult inpatient hospice services.	Spatial analysis	Area deprivation	Hospices	**Access issues:** Geographic accessibility **Key findings:** 41% of wards in the North West where access was poor and demand relatively high were relatively highly deprived.

*HCP* Healthcare Professionals

### Findings

There was insufficient evidence to synthesise data under the seven separate stages of candidacy. For example, there was little evidence of how ‘permeable’ services were (the ease with which they can be accessed and the degree of cultural alignment required), nor in the extent to which offers of care are accepted or refused. Instead, four broader themes were generated, within which the concepts of candidacy were captured: identifying needs; taking action; local conditions; and receiving care. In the original model, the first stage of candidacy was an individual’s ‘identification’ of their own candidacy for healthcare, followed by their ‘navigation’ into, and ‘appearance’ at, a service. The broader themes generated from this synthesis do not adhere to the same chronology, reflecting the uncertainty within palliative care as to whether a patient, family member, or professional would first identify a need for care and initiate a conversation.

### Identifying needs

While no studies explicitly sought to examine differences in need, or how people identify need, for palliative care between socioeconomic groups, there were some indications that needs could vary. One study found that financial and housing issues, for example, were greater in the last year of life for working class than middle class people [41]. One hospice reported a greater number of visits to patients in socially deprived boroughs of London, than those in high income areas [49]. In another study, overall quality of care received by patients at the end of life was perceived to be worse in the 20% most socially deprived areas of England, than in the least deprived areas [7]. Despite these indications of potentially greater need, or unmet need, for palliative or end-of-life care there was little exploration about how patients and professionals may assess need for care differently depending on a patient’s socioeconomic position, a fundamental component to the candidacy model.

Evidence on awareness or attitudes towards death and dying among patients from different socioeconomic groups was minimal, over 20 years old, and contradictory, making it hard to synthesise; the higher quality study found that patients in a more advantaged class were more accepting and aware of death and dying [56]. Only one qualitative study considered attitudes to hospice care, finding no examples of differences between social classes [53]. There is a lack of evidence, therefore, that those in a more socioeconomically disadvantaged position would be less likely to recognise they had a need for palliative or end-of-life care.

With regards to how professionals assessed patient need, one study found that ‘working class’ patients were more likely to feel a general practitioner has less time to talk [41]. Another suggested that so-called lifestyle factors that the authors associated with socioeconomic disadvantage, may lead to nurses downgrading patient reports of pain [59]. This highlights the potential for bias in how patients are assessed. Encouragingly, however, a study of GP palliative care training in Wales found no evidence of differences in training across socioeconomic areas [48].

### Taking action

Whether someone receives the care they need depends on the abilities of patients, families, and healthcare providers to take steps to secure that care. There was some evidence to suggest socioeconomically disadvantaged groups and communities may have fewer informational resources to help navigate this process. This evidence showed patients who were more socioeconomically disadvantaged being less likely to recognise the phrase palliative care or correctly understand the role of Macmillan nurses [54], showing less desire for information or preferring to “passively” acquire it [53], finding it difficult to ask for information [52], and misunderstanding the role of an out of hours palliative care service [61].

Although families can facilitate access to care, only two studies closely examined the relationship between socioeconomic position and families. One found that patients often relied “on their most forceful members, particularly children of higher social class” to achieve access to a hospice bed ([53], p.108). Additionally, Johnson et al. [43] found that household income of carers was not related to access to palliative care, but higher qualifications were, particularly having a degree. The ability to navigate care successfully may, therefore, have a stronger link with having a highly educated, possibly younger, care advocate.

It is not necessarily that patients and carers experiencing disadvantage do not ask for care, but that sometimes requests appear to go unheard. In one study of Bangladeshi carers in East London, a carer in precarious social circumstances reported not receiving formal support even after they “begged the authority for help” ( [57], p.126), and only received help after a fire broke out in their kitchen. Cartwright [41] also found that more working class than middle class patients had difficulties overcoming barriers to care related to housing, sometimes financially driven.

### Local organisation

While the organisation of services could be a barrier to access, it was unclear whether such barriers impact patients differently depending on socioeconomic circumstances [52, 61]. One qualitative study reported that patients in a disadvantaged social class assumed they have access to a hospice bed when they are dying, an assumption in contrast to the reality of scarce resources and limited referral options available to them [53]. Local context is clearly important in understanding the impact of service availability. While most hospices (77.1%) serve mixed deprivation areas, more operate in affluent areas (15.7%) than in deprived areas (7.1%) [55]. Some regions in England have a higher proportion than others of socially deprived areas over 30 min drive from a hospice inpatient unit, indicating that the relationship between area deprivation and geographic accessibility is not consistent throughout the country [51, 62].

Regional differences are also evident in the length of time between referral to hospices and death. The time spent under hospice care in the Midlands or South of England is longer on average than in the North of England – a more disadvantaged region on average [37].

Where inequities in access do exist, they are unlikely to only result from differences in service availability: a study of a single hospice at home service delivered to two socioeconomically distinct areas found increasing area deprivation was associated with lower referral rates [63], suggesting that availability could not fully explain inequities in referrals in that instance.

### Receiving care

#### Receiving care from generalist palliative and end-of-life care providers

The use of hospital or primary care services does not necessarily mean an individual has received generalist palliative care. However, these services have the capacity to provide generalist palliative care, making it appropriate to consider the association between socioeconomic position and receipt of these services. Using hospital services, particularly emergency care, at the end of life is consistently associated with socioeconomic disadvantage [8, 15, 58]. The evidence regarding primary care referrers was more mixed, with one study finding that both patients with financial difficulties and those who paid for health services were more likely to be frequent attenders of GP services in the last year of life [47]. A study of a hospital at home service for people in the last 2 weeks of life found that patients tended to live in less deprived areas, although there were no statistically significant differences in referrals by social class [46]; a third older, smaller study also found no social class differences in the use of GP services or in nurse visits towards the end of life [41].

#### Receiving care from specialist palliative care providers

There was an overall trend for findings to suggest no evidence of differences in receipt of specialist palliative care between socioeconomic groups, although this may depend on a number of factors [7, 8, 35, 36, 39, 41, 42, 44, 64]. For example, there was a slight trend for findings based on survey data to suggest no evidence of a difference in receipt of specialist palliative care between socioeconomic groups [7, 35, 36, 39, 64]. A similar pattern was found for findings based on individual measures of socioeconomic position [35, 36, 42, 64], and from studies using national representative samples [7, 35, 36, 41, 64].

In contrast, findings based on routinely collected data [37, 44, 63] or which used local data [38, 44, 63] tended to report socioeconomic inequities in receipt of specialist palliative care.

## Discussion

This aims in this review were to understand the extent to which socioeconomic position influences access to palliative and end-of-life care in the UK and explore how factors relating to socioeconomic position influence access to this care. An additional aim was to explore the usefulness of the candidacy model for understanding socioeconomic inequities in access to palliative and end-of-life care.

This study reiterates the finding that socioeconomically disadvantaged populations are more likely to receive hospital-based care at the end of life, and that there is a lack of evidence regarding access to and use of services that might be providing generalist palliative care in the community [7, 10]. The review findings did not suggest a consistent or clear narrative regarding the relationship between socioeconomic position and receipt of specialist palliative care in the UK, with many studies finding no evidence of differences in receipt of care between socioeconomic groups. Finding an absence of evidence does not preclude there being socioeconomic inequities in access to palliative care in the UK. However, it indicates there is currently very poor understanding within the UK of the extent to which these exist. While it is possible to draw on evidence from the United States, Canada, and Australia, which suggests an overall trend towards individual socioeconomic disadvantage being associated with lower odds of using specialist palliative care [10], further research in UK should look to clarify where and when inequities in receipt of care occur.

Ascertaining whether differences or similarities in receipt of palliative care are inequitable or equitable requires better understanding of the relationship between socioeconomic position and need for palliative care, particularly population level need. This issue has been identified in earlier studies and was reiterated again in the findings from this review [10, 13]. Building on this evidence base, the findings from this review point towards specific evidence gaps within the UK context concerning the relationship between socioeconomic position, how need - or ‘candidacy’ - for palliative care is assessed, and the organisation of care.

Ideally, need for palliative care is determined by assessing a range of different patient characteristics, such as physical or emotional symptoms, spiritual distress, preferences, and prognosis [65]. In practice, other factors relating to healthcare professionals and local context are also often taken into account [66, 67]. While acknowledging the challenges of defining need for palliative care, without a clear conception of need, it is difficult to conclude whether access to care is inequitable [68]. Given that pressures on healthcare services are often greater in more socioeconomically disadvantaged areas [69, 70], understanding whether external service pressures are considered an appropriate indicator of ‘need’ for care, and how this influences access to palliative care, may be critical in understanding why and when socioeconomic inequities arise. Despite this, few studies in this review explored how patients and professionals assess need in the context of socioeconomic disadvantage, or the relationship between this and organisational context.

The findings from this review, and the theoretical arguments proposed within the candidacy model of access, position access as an interaction between people and healthcare services that is contextualised by how services are organised locally. This is important for answering the question of ‘how’ different factors influence access to palliative and end-of-life care. On the one hand, there was suggestion in this review of potential differences in information resources held by patient and families, as well as in understanding of health and healthcare at the end of life. This is similar to health literacy-related barriers to access identified in reviews of international evidence on palliative care and socioeconomic position [11]. However, there were also indications that professionals in socially deprived areas had less time to talk or did not respond to requests for help. This calls for attention on the interaction between communication, information, and service resourcing, and its influence on inequities in access.

Local organisation of services is considered an important influence on assessing candidacy for care among socioeconomically disadvantaged groups [16, 27]. Most findings in this review relating to how services are organised focused on geographical accessibility of services. While compared to many other countries, the UK has well-developed palliative care services [23], the evidenced documented in this review suggests a potential regional bias in service availability favouring more advantaged areas. However, beyond geographical availability, equitable access to palliative and end-of-life care is likely to require changes to traditional models of care, how services are delivered, and to inter-professional working relationships [17, 71]. Future studies would benefit from expanding on geographical accessibility to consider how these other aspects to service organisation may result in different patterns of access for socioeconomic groups in UK settings. Population health approaches to palliative care could be used to identify population need and facilitate equitable responses, considering the local population and organisation context [17, 72, 73].

A further evidence gap in the UK concerns the role of mistrust and stigmatisation in driving inequities in access. Evidence from other countries of patient mistrust in services and the stigmatisation of patients from disadvantaged backgrounds was not widely replicated in studies included in this review [18, 74]. There is a need in the UK to better understand how these issues may be experienced by people who are disadvantaged not just in their socioeconomic position but across multiple characteristics, including age, ancestry, and gender. Of the studies synthesised in this review, only one explicitly considered the relationship between socioeconomic position and ancestry [57], but there was little evidence relating to the intersection of gender and socioeconomic factors, despite family caregiving in palliative care being highly gendered [75]. Mistrust of services and stigma have been documented in studies of palliative care access for people experiencing homelessness in the UK [76]. Research with homeless populations was not included in this review, because the specific services (hostels, harm reduction services) tailored to people experiencing homelessness mean their experiences of accessing palliative care are likely to be specific to that population group [77]. Additionally, several recent systematic reviews had already been conducted for that topic [76–78]. However, future studies may want to explore whether mistrust of services is an experience relevant to other populations experiencing socioeconomic disadvantage in the UK.

### Assessing the candidacy model of access

The candidacy model provided a useful lens through which to view the evidence, largely as it made clear the gaps in evidence described above. In focusing on patient-professional interactions, access is posited as a phenomenon generated through social interactions influenced by context, rather than something that an individual does. This focus on social interactions complements the philosophy of palliative care, where compassion, communication and building trusting relationships are paramount [79], justifying the use of the model in this review.

There were some limitations to using this model in this review. The suggestion within the candidacy model that access begins with a person identifying their own need for care may be inappropriate within palliative care, where referrals tend to be initiated by healthcare professional rather than by patients [65], although some clinicians are reluctant to do this [80–82]. The lack of focus on the influence of structural factors on equitable access is a further limitation of the model, and one already noted in other research [27]. Future uses of candidacy within palliative care may benefit from incorporating concepts from other theories or models, including those that explicitly reference high-level political, economic, and social structures underpinning socioeconomic inequities [17, 73].

### Limitations

Any analysis of access to palliative care is substantially hindered by the lack of accurate accounting for differences in need for palliative care, long identified as an issue [10, 68]. The limited amount of evidence for some of the seven original stages in the candidacy model meant it was necessary to synthesise the data under broader themes, although these still reflected the sentiments of original model constructs. The long time span covered in this review (1990 to 2020), and that most studies’ primary focus was not socioeconomic position, also made it difficult to synthesise findings. As the evidence base for this topic expands, future reviews could aim to synthesise studies with a primary focus on socioeconomic factors, in particular those using qualitative methods where having rich and detailed data would strengthen the synthesis.

### Implications

The findings of this review imply that attempts to address inequities in access will require knowledge and action across many different areas. For example, raising awareness of palliative care amongst those experiencing socioeconomic disadvantage is unlikely to be sufficient to generate access, without understanding and addressing barriers related to how services are organised or needs are identified. There are many evidence gaps and areas of uncertainty within the UK context that further research could address, including which service models are more effective are reaching socioeconomically disadvantaged populations, the influence of socioeconomic factors on how needs are assessed, or the extent to which mistrust of services and stigma is a barrier to access.

## Conclusion

There is a not a clear and consistent narrative in UK literature regarding the relationship between socioeconomic position and access to palliative and end-of-life care. While there is some evidence indicating socioeconomic differences in informational resources to help navigate the process of accessing care, there is less understanding about which service models are effective at reducing inequities, or how socioeconomic factors affect how patients, families, and professionals assess needs. The candidacy model of access is applicable to palliative and end-of-life care although other concepts may need to be incorporated to capture the full range of factors influencing access to care for those experiencing socioeconomic disadvantage.

## Supplementary Information

**Additional file 1: Supplementary material 1.** Stage 1 of a narrative synthesis (developing a theoretical understanding). **Supplementary material 2.** Medline search strategy. **Supplementary material 3.** Study eligibility. **Supplementary material 4.** Data extraction form. **Supplementary material 5.** Quality appraisal.

## Data Availability

As this was a review of existing literature, all data and materials came from published materials already in the public domain. Links to the original publications can be found in the references.
