# Correction to: Exploring socioeconomic inequities in access to palliative and end-of-life care in the UK: a narrative synthesis

**DOI:** 10.1186/s12904-021-00887-z

**Published:** 2021-12-12

**Authors:** Maddy French, Thomas Keegan, Eleftherios Anestis, Nancy Preston

**Affiliations:** 1 Division of Health Research, Lancaster University, Lancaster, UK; 2 Lancaster Medical School, Lancaster University, Lancaster, UK

**Correction to: BMC Palliative Care 20, 179 (2021)**

**https://doi.org/10.1186/s12904-021-00878-0**

Following the publication of the original article [1], the author reported that a phrase in the abstract section was omitted during production process.

The sentence currently reads:

Efforts inequities in access to palliative and end-of-life care require comprehensive understanding about the extent of and reasons for inequities.

The sentence should read:

Efforts **to tackle socioeconomic** inequities in access to palliative and end-of-life care require comprehensive understanding about the extent of and reasons for inequities.

The original article has been updated.
