# Supplementary material for: Exploring socioeconomic inequities in access to palliative and end-of-life care in the UK: a narrative synthesis
Source: BMC Palliat Care. 2021 Nov 21;20:179. doi: 10.1186/s12904-021-00878-0 (PMC8606060; doi:10.1186/s12904-021-00878-0)
Supplement: Supplementary file 1 — Additional file 1: Supplementary material 1. Stage 1 of a narrative synthesis (developing a theoretical understanding). Supplementary material 2. Medline search strategy. Supplementary material 3. Study eligibility. Supplementary material 4. Data extraction form. Supplementary material 5. Quality appraisal. [file 12904_2021_878_MOESM1_ESM.docx]

Supplementary Materials

1. Stage 1 of a narrative synthesis (developing a theoretical understanding)
2. Medline search strategy
3. Study eligibility criteria
4. Data extraction form
5. Quality appraisal scores

Supplementary material 1: Stage 1 of a narrative synthesis (developing a theoretical understanding)

| Stage of access | Factors that shape access | Factors in the original model | Additional hospice-related factors |
| --- | --- | --- | --- |
| Identification of candidacy | Acceptance of death  Acceptance of terminal illness Awareness of death or dying Help-seeking behaviours Normalisation of symptoms  Patient or family downgrading symptoms  Lifetime experience of ill health | - Services more likely to be used as a series of crises. - Help-seeking likely to occur in response to specific events rather than planned. - Symptoms may be “downgraded” - Lack of positive conceptualising of health - Normalisation of symptoms due to consistent experience of ill health in self and in community/family. | Differences in acceptance of death or awareness of death may effect whether someone sees themselves as a suitable candidate for hospice care. It may effect desires to make plans about an individual’s death, making end of life discussions more difficult. |
| Navigation | Knowledge or awareness of hospice  Knowledge or awareness of services  Available resources – practical, social, financial, mobile (e.g. transport) | - Awareness of services on offer - Mobilising practical resources (e.g. time off work, financial support) - Mobilising social resources (e.g. support at home, support for transport) | Differences in awareness of hospice services may lead to misunderstandings, and therefore rejection of referrals. Some people may have less access to advocates (social support) who support people to ask for help or to articulate needs. May be processes to overcome some resources problems but perception of these problems may be enough to deter someone from accepting hospice care. |
| Permeability of services | Perception of hospice/services Hospice culture  Attitudes towards death or dying  Service availability  Type of services available  Experiences of gatekeepers (e.g. referrers – GPs, nurses etc) | - The way services are organised affects the ease with which people can access them (or how ‘permeable’ a service is) - Some services require referrals, certain symptoms, a certain diagnosis, in order to access them - Less permeable services ‘demand a higher degree of cultural alignment between themselves and their users’. Comfort with organisational values of service and referrer important. - Satisfaction following previous encounters may affect later experiences | Referral typically needed to access specialised hospice care. Diagnosis and symptoms are required for a referral to be generated. A hospice death or a ‘good’ death may not be culturally aligned with the preferences of everyone in society. |
| Appearances | Articulating symptoms/issues/needs  Verbal activity  Demand for care or support  Persistence | - People make ‘claims’ for their right to access services - Patients required to formulate and articulate issue in a way that aligns with clinical assessment - Social distance between clinician and patients may make this harder | Palliative care may differ slightly in that a patient will already be known to a clinician and unlikely to be ‘presenting’ for the first time with end of life symptoms. |
| Adjudication | Referral judgements/decisions  Criteria for referrals  Clinician knowledge of services  Clinician engagement with services  Clinician assumptions about patients ability to benefit | - Clinicians have to make judgement calls about who to refer and who to accept into a service - These decisions are strongly linked to relationships with other clinicians, local resources and capacity - Clinicians may ask how likely a patient is to benefit from an intervention (sometimes this may have social criteria) | Clinical judgements made on who is likely to benefit from hospice (e.g. symptoms, diagnosis)  In disadvantaged areas, clinicians have less time to engage with services in local area and build their knowledge of local palliative care support available. Or they may look to offload work by increasing referrals. |
| Offers and resistance | Refusal of care Resistance to care Perception of hospice  Family rejection of care | - Patient (or family) may resist offers of referral - Resistance may be due to patient’s identification of candidacy (see above) or perception of service etc | Rejection may be due to differences in acceptance of terminal phase of illness, desire for curative care, attitude towards death and dying (see above) |
| Operating conditions | Local resources  Local capacity Availability of services Joined up care Fragmented care | - Locally specific influences on interactions between patients and clinicians - Fragmented complex systems harder to navigate for disadvantaged groups - Resource scarcity may prevent referrals (tied to adjudication) | Always a limit to number of people who can receive care from hospice. In reduced circumstances, disadvantaged groups more likely to miss out due to above reasons. |

Supplementary material 2: Medline search strategy

| S13 | S6 AND S9 AND S12 |
| --- | --- |
| S12 | S10 OR S11 |
| S11 | (MH "Health Care Quality, Access, and Evaluation") OR (MH "Health Services Accessibility") OR (MH "Referral and Consultation") OR (MH "Patient Acceptance of Health Care") OR (MH "Treatment Refusal") OR (MH "Referral and Consultation") OR (MH "Gatekeeping") OR (MH "Health Care Delivery") |
| S10 | TX ( refer* OR experience* OR access OR Utilisation OR utilization OR barrier* OR facilitat* ) OR TX ( service* N5 (pattern* OR provision OR delivery OR supply OR distribution OR availability OR use OR uptake) ) OR TX ( perception* N5 (hospice OR palliative OR terminal OR death OR dying OR "end of life" OR end-of-life) ) OR TX ( awareness* N5 (hospice OR palliative OR terminal OR death OR dying OR "end of life" OR end-of-life) ) OR TX ( attitude* N5 (hospice OR palliative OR terminal OR death OR dying OR "end of life" OR end-of-life) ) OR TX ( accepta* N5 (hospice OR palliative OR terminal OR death OR dying OR "end of life" OR end-of-life) ) OR TX ( knowledge* N5 (hospice OR palliative OR "end of life" OR end-of-life) ) OR TX ( afford* N5 (hospice OR palliative OR "end of life" OR end-of-life) ) OR TX ( demand* N5 (hospice OR palliative OR "end of life" OR end-of-life) ) OR TX ( availability N5 (hospice OR palliative OR "end of life" OR end-of-life) ) OR TX ( ("decision making" OR decision-making) N6 (palliative OR hospice* OR "end of life" OR end-of-life) ) OR TX ( (Myth* adj5 (palliative or “end of life” or hospice OR end-of-life)) OR (Misconception* adj5 (palliative or “end of life” or hospice OR end-of-life)) ) |
| S9 | S7 OR S8 |
| S8 | (MH "Poverty") OR (MH "Social Class") OR (MH "Socioeconomic Factors") |
| S7 | TI ( socioeconomic* N2 disparit* OR disadvantage* OR inequality* OR inequit* OR equit* OR depriv* ) OR AB ( socioeconomic* N2 disparit* OR disadvantage* OR inequality* OR inequit* OR equit* OR depriv* ) OR TI ( Social N2 disparit* OR disadvantage* OR inequality* OR inequit* OR equit* OR depriv* OR determinant* ) OR AB ( Social N2 disparit* OR disadvantage* OR inequality* OR inequit* OR equit* OR depriv* OR determinant* ) OR TI ( “medically uninsured” OR “social class” OR “social capital” OR poverty OR low-income OR discriminat* OR income ) OR AB ( “medically uninsured” OR “social class” OR “social capital” OR poverty OR low-income OR discriminat* OR income ) |
| S6 | S1 OR S1 |
| S2 | (MH "Hospice Care") OR (MH "Hospices") OR (MH "Terminal Care") OR (MH "Hospice and Palliative Care Nursing") OR (MH "Palliative Medicine") OR (MH “Attitude to Death”) |
| S1 | TI ( hospice* or palliat* or "end of life" or "end-of-life" or dying or (terminal* N6 ill*) or (terminal* N6 care*) ) OR AB ( hospice* or palliat* or "end of life" or "end-of-life" or dying or (terminal* N6 ill*) or (terminal* N6 care*) ) |

Supplementary material 3: Study eligibility

|  | ***Inclusion criteria*** | ***Exclusion criteria*** |
| --- | --- | --- |
| ***Type of evidence*** | - Peer-reviewed journal articles (original data – e.g. population studies, qualitative data) - Grey literature reports - Commentary/opinion articles based on primary data findings | - systematic reviews - abstracts - opinion/commentary pieces not based on primary data findings |
| ***Study population*** | - Adult (+18) patients - Have an advanced progressive illness - Described in socioeconomic terms OR - The families of these patients - Health and social care workers treating these patients - Hospice organisations providing care to these patients | - patients under 18 - currently homeless - currently in prison |
| ***Study topic*** | Access to SPC, including:   - Awareness of hospice or palliative care (HPC). Alternative terms: end of life care, terminal care - Availability and quality of resources (patient: practical, social, family, financial, advocacy) (services: availability, capacity) - Attitudes towards or perception of HPC - Attitudes towards death, dying, or terminal illness - Referral experiences/decisions/criteria - Communication (patient: articulation, verbal activity) (clinician: prognosis, information sharing) - Relationship between patients/families and HPC staff or referrers - Stigma - HPC values or culture - Gatekeeping - Demanding or help-seeking behaviours - Resistance to/refusal of care - Joined up or fragmented care - Normalisation or downgrading of symptoms - Utilisation or receipt of care | - Place of death - Advanced care planning - Supportive care |
| ***Study measures*** | A measure of socioeconomic position should be explicitly mentioned in either the title or abstract of the study. Socioeconomic position is measured by:   - Area/postcode deprivation - Income (household/individual) - Education - Employment (prior illness) - Social Class - Social capital - Subjective measures (e.g. perception of socioeconomic position)   OR   - Any other measure described by study authors as a measure of socioeconomic position/disadvantage/deprivation/poverty | Studies that only report   - Uninsured patients (e.g. Medicaid) - Ethnicity - Age - Gender |
| ***Language*** | English language | Language other than English |
| ***Setting*** | UK | Countries outside the UK |
| ***Timescale*** | Published 1990 or later | Published before 1990 |

Supplementary material 4: Data extraction form

Complete for all studies

Complete for quantitative findings

| **Author(s)** |  |
| --- | --- |
| **Date** |  |
| **Title** |  |

| **Study type** |  |
| --- | --- |
| **Study design** |  |
| **Type of care** |  |
| **Study setting** |  |
| **Aims** |  |
| **Analysis method** |  |

| **Study population/**  **perspective** |  |
| --- | --- |
| **Sample size** |  |
| **Participant characteristics** |  |

| **Measure(s) of access (details)** |  |
| --- | --- |
| **Measure(s) of access (simplified)** |  |
| **Measure(s) of socioeconomic position** |  |
| **Effect size (incl. significance)** |  |

| **Effect modifiers**  **/ confounders** |  |
| --- | --- |
| **Adjusted effect size (either individual or total)** |  |
| *Add more rows if needed* | |

| **Key findings** |  |
| --- | --- |

Supplementary material 5: Quality appraisal

| Study | Abstract/ title | Introduction / aims | Method / data | Sampling | Data analysis | Ethics  / bias | Results | Transferability | Implications / usefulness | Total | Reasons for score deduction |
| --- | --- | --- | --- | --- | --- | --- | --- | --- | --- | --- | --- |
| Addington-Hall and Altmann, 2000 | 4 | 4 | 4 | 3 | 3 | 1 | 4 | 4 | 3 | 30 | Sample size from each district not justified; limitations of using social class not discussed (e.g. relationship between age and social class and death certificate); implications for practice not explicitly outlined |
| Addington-Hall et al., 1998 | 4 | 4 | 4 | 4 | 4 | 3 | 3 | 4 | 3 | 33 | Ethical issues not discussed in detail although potential bias of recollection was discussed; findings relating to social class not reported in text; implications and further research not discussed in relation to social class differences |
| Allsop et al., 2018 | 4 | 4 | 4 | 4 | 4 | 2 | 4 | 4 | 4 | 34 | Ethics mentioned but not discussed in depth |
| Barclay et al., 2003 | 4 | 3 | 4 | 4 | 4 | 1 | 4 | 4 | 4 | 32 | More discussion of ethics and risk of bias warranted. |
| Buck et al., 2018 | 4 | 3 | 4 | 4 | 2 | 3 | 2 | 3 | 3 | 28 | Aims, objectives, research questions unclear; analysis not adjusted for other variables; tables related to deprivation and access difficult to interpret; no percentages for supply and demand provided for hospice at home service figure; thematic analysis results not reported in detail |
| Burt et al., 2010 | 4 | 4 | 4 | 4 | 4 | 3 | 4 | 4 | 4 | 35 | Light on discussion of ethical issues |
| Campbell et al., 2010 | 4 | 4 | 3 | 4 | 4 | 2 | 4 | 3 | 4 | 32 | Method of using population data might be limited in answering question of 'how' SEP influences access; no mention of ethical approval or general ethical issues, although authors mention potential bias of population data. |
| Cartwright, 1992 | 3 | 2 | 3 | 3 | 2 | 1 | 4 | 2 | 2 | 22 | Unclear in abstract if interviews collect quantitative or qualitative data; previous relevant literature e.g. Black Report, not referenced in introduction; survey methodology might not be adequate to address research questions relating to experiences and life circumstances before death; middle and working class risks oversimplifying occupational classes; few details on setting or demographics of respondents; statistical tests not mentioned and ambiguous descriptions of statistical significance; no mention of ethics or bias related to using recollection; minimal description of England or district context or about respondents other than they were randomly sampled; does not discuss practice implications or recommend research. |
| Clark, 1997 | 1 | 2 | 1 | 3 | 2 | 1 | 2 | 2 | 3 | 17 | Commentary piece based on primary data results. Few details given on the methods, explanation of results and no justification about combination of Lambeth and Southwark in one category and other boroughs in individual category. |
| Dixon, et al., 2015 | 4 | 4 | 3 | 3 | 4 | 2 | 3 | 4 | 4 | 31 | No traditional abstract format but very extensive executive summary; only significant results reported; survey method relies on respondents correctly remembering services received; not clear who was eligible to be sampled from the bereavement survey. |
| Fergus et al., 2010 | 4 | 3 | 4 | 3 | 4 | 1 | 4 | 3 | 3 | 29 | No details on individual participant characteristics. Ideas for further research not explored. |
| Fisher et al., 2016 | 1 | 3 | 4 | 4 | 3 | 2 | 3 | 3 | 3 | 26 | No abstract; no research question; few details provided on context and setting; potential bias of record system discussed but not in detail; further research discussed but impact on practice not clear; statistical significance not reported |
| Gatrell and Wood, 2012 | 4 | 4 | 4 | 4 | 4 | 3 | 4 | 3 | 4 | 34 | Limitations about using cancer deaths as a proxy measure of need not discussed; transferability limited |
| Grande et al., 2002 | 4 | 4 | 4 | 4 | 3 | 2 | 4 | 4 | 3 | 32 | Some uncertainty in methods (e.g. unclear if the proportion of patients who received no input from services was different across SE groups. They appear to be excluded from the analysis). Could have chosen alternative model or discussed alternatives (e.g. a zero inflated count data model); Some phrases were not very clear e.g. "deprivation scores formed clusters in the data set"; minimal discussion about ethics or bias. |
| Gray and Forster, 1997 | 3 | 4 | 4 | 4 | 2 | 2 | 4 | 3 | 3 | 29 | No detail on how data collected in abstract; analysis described but statistical tests not named; ethical permission named but no ethics or bias issues reported or discussed; more detail on setting and deprivation/healthcare context warranted; practice implications not discussed |
| Hanratty et al., 2012 | 4 | 4 | 4 | 3 | 4 | 2 | 4 | 3 | 2 | 30 | Few details on how clinicians were sampled; as SEP not reported with individual quotes or experiences, usefulness is limited in this context; not clear who invited participants and the potential effect of this. |
| Hanratty et al., 2008 | 4 | 4 | 3 | 3 | 2 | 1 | 2 | 2 | 2 | 23 | No justification why rapidly deteriorating patients were only sampled rather than all patients with cancer or heart failure in last year of life; data linkage process unclear; actual figures not reported in results and few CIs and no p values provided; |
| Hanratty, Jacoby, and Whitehead, 2008 | 4 | 4 | 4 | 4 | 4 | 2 | 3 | 4 | 3 | 32 | Limited mention of ethics, some discussion of potential bias; results were logical but no explanation why only some outcomes were reported (e.g. GP use and not district nurse visits); some data missing to support finding related to financial strain and receiving illness related benefit. |
| Johnson et al., 2018 | 3 | 4 | 4 | 4 | 4 | 2 | 3 | 4 | 4 | 32 | Not clear which sample the abstract results refer to; potential discrepancy (or lack of clarity) between text and table significance results; no mention of potential bias of measures and question put to respondents and respondent bias. |
| Kessler et al., 2005 | 4 | 4 | 3 | 3 | 3 | 1 | 3 | 2 | 3 | 26 | Contradictory information about the number of interviews; framework analysis method described but not in detail; ethical permission not mentioned, although bias of proxy accounts explored; barriers to control not thoroughly explored; minimal details about participants or Bristol hospice, healthcare, or deprivation setting. Some outcomes might have been better addressed using survey data than qualitative; few quotes provided to back up findings; no further research suggested |
| Koffman et al., 2007 | 4 | 4 | 4 | 4 | 3 | 1 | 3 | 4 | 4 | 31 | Lacking detail on free text coding technique; unclear if patients were interviewed by hospice staff and the potential impact of this on their knowledge of hospice; range of IMD does not include anyone in top 30% deprivation areas - implications not discussed. |
| London Cancer Alliance, PallE8 and Marie Curie (London Cancer Alliance) 2015 | 4 | 4 | 2 | 3 | 2 | 2 | 3 | 3 | 3 | 26 | Few details given on the methods and how specialist palliative care services were identified, who in the service filled out the audit template; audit template not provided |
| Marie Curie Cancer Care and the Bevan Foundation (Marie Curie) 2014 | 2 | 3 | 2 | 3 | 2 | 2 | 4 | 3 | 4 | 25 | No abstract or overall summary of findings at outset; Methods of analysis and choice of analysis not reported in detail; Sampling strategy for section "receiving spc" not clearly outlined in text; minimal discussion of potential bias or ethical issues; limited context given |
| Seale, et al., 1997 | 4 | 3 | 4 | 3 | 3 | 1 | 4 | 4 | 2 | 28 | No clear objectives or research questions; choice of districts not justified; sample size not justified; not clear why just subset of data was analysed; some data appear to be missing but not reported in study e.g. analysis on social class was only on ~400 sample; ethics not discussed and potential for bias recollection not discussed; no explicit implications for practice described and no further research suggested. |
| Spruyt, 1999 | 4 | 3 | 2 | 3 | 1 | 2 | 2 | 3 | 2 | 22 | Described as ethnographic but methods suggest semi-structured interviews, although no details provided about analysis methods and little consideration of ethics. |
| Walsh and Laudicella, 2007 | 4 | 4 | 4 | 4 | 3 | 1 | 2 | 4 | 4 | 30 | Costs on average not clearly described (costs over what time) |
| Wilson, 2009 | 3 | 4 | 2 | 2 | 4 | 2 | 4 | 3 | 2 | 26 | Full results not included in the abstract; no info on where participants were sampled from; no discussion about whether drink driving a fair indicator of lower socioeconomic status; potential bias of nurse managers distributing leaflets not discussed; ethical issues of not telling nurses what true aim of study not discussed; small sample size not discussed. |
| Wood et al, 2009 | 3 | 4 | 3 | 4 | 3 | 2 | 3 | 2 | 4 | 28 | Results not included in abstract; not clear why drive times over 30 discounted particularly given rurality of area; actual method of analysis not specified; results were logical although more detail on the deprived wards with high need and low access warranted. |
| Rees-Roberts, M. et al. 2019 | 4 | 3 | 4 | 3 | 4 | 2 | 4 | 3 | 4 | 31 | Few details on bias or ethical issues. More could have been described about the wider UK hospice/palliative care context to help with transferability. |
